# Low haemoglobin predicts early mortality among adults starting antiretroviral therapy in an HIV care programme in South Africa: a cohort study

**DOI:** 10.1186/1471-2458-10-433

**Published:** 2010-07-23

**Authors:** Elizabeth C Russell, Salome Charalambous, Lindiwe Pemba, Gavin J Churchyard, Alison D Grant, Katherine Fielding

**Affiliations:** 1 London School of Hygiene and Tropical Medicine, London, UK; 2 Aurum Institute, Johannesburg, South Africa

## Abstract

**Background:**

Antiretroviral therapy (ART) has dramatically reduced morbidity and mortality among people with HIV infection; however, mortality after the start of ART is high in resource-limited settings. We investigated risk factors for mortality among adults starting ART in a multi-clinic community programme in South Africa.

**Methods:**

Cohort of adults starting ART at 27 clinics between February 2005 and June 2006, followed to 31^st ^March 2007. Kaplan-Meier survival estimates were used to describe overall mortality. Shared frailty Cox regression was used to identify baseline risk factors for early mortality.

**Results:**

Among 1350 participants (median age 35.5 years, 60% female, median CD4 count 83/μL [interquartile range (27 - 147)], median follow-up 13.4 months), there were 185 deaths, overall mortality rate 13/100 pyrs; for 0-3, 3-9 and >9 months from ART start mortality rates were 24, 13 and 6/100 pyrs respectively. 43% of the deaths were in the first 3 months of treatment. Risk factors for mortality in univariable analysis were baseline CD4 count, viral load, haemoglobin and body mass index, in multivariable analysis adjusting for age and gender, only CD4 count and haemoglobin remained independently associated with proportional hazards not being satisfied for haemoglobin. Adjusted hazard ratios (aHR) for participants with haemoglobin <8, 8.1-9.9, >11.9(f)/12.9 (m) g/mL were 4.99, 3.05 and 0.12 respectively comparing to 10-11.9 (f)/12.9 (m)g/mL in the first 3 months of ART. aHRs for CD4 counts were 0.40, 0.38 and 0.34 for 50-99, 100-200 and >200/μL comparing to <50/μL.

**Conclusions:**

The high mortality rate in the first 3 months underlines the need for earlier HIV diagnosis so that ART can be initiated earlier. Low haemoglobin and low CD4 count are both strong predictors of mortality, and could be used to identify individuals at high risk who might benefit from intensive case management.

## Background

South Africa is estimated to have 5.7 million HIV-positive individuals out of a total population of 48.6 million [1] and has more people living with HIV/AIDS than any other country worldwide [2] which is a considerable challenge for health care systems and communities across the country. A key component of a suitable and extensive response to this growing epidemic, especially in communities most affected by HIV, is combination antiretroviral therapy (cART) [3] and it is estimated that 889,000 adults (less than 49 years old) and children in South Africa are in urgent need of this treatment [4].

Although treatment programmes have been scaled up and increased in number they still face challenges. Studies in Sub-Saharan Africa show that one of these challenges is that mortality in these programmes is much higher in the first three months [5-9]. One of the reasons for this is that despite better availability of ART, people are still diagnosed late and thus start ART when they already have late stage disease[9]. Identifying those at highest of early mortality risk could help target interventions to improve survival.

The data for this study were collected from a large community based programme set in clinics run by NGOs or private practitioners in five provinces of South Africa. The clinics provide standardised HIV care and treatment through a programme implemented by the Aurum Institute. We describe early mortality and loss to follow-up, and risk factors for mortality in this setting.

## Methods

### Study design, setting and population

This was a prospective cohort study examining routinely collected clinic data from an observational cohort of adult patients to examine risk factors for early mortality. The clinics contributing data to the study were in urban and peri-urban sites in five provinces of South Africa (Gauteng, Limpopo, Free State, Mpumalanga, and North West). The clinics were either run by a non-government organisation (NGO), or private practitioners. Training, clinical and financial support, drug procurement, and administration and monitoring support were provided by the Aurum Institute. This programme provided free antiretroviral treatment to patients who did not have medical insurance and were unable to pay for their treatment themselves. The organisation of the programme as it was originally developed in an industrial setting has been described elsewhere [10].

The clinics followed the National South African HIV treatment guidelines for participants starting ART: either CD4 <200/μL or classified as in WHO stage 4. The first line regimen given to the majority of patients was stavudine, lamivudine, and either nevirapine or efavirenz. Patients starting ART attend monthly to collect medication; they have a clinical review and blood taken for full blood count and liver function tests at six weeks, three months and three monthly thereafter; and CD4 count and viral load at six weeks, six months and six monthly thereafter.

Patients were included in this analysis if they were 18 years or older when initiating ART treatment, if they were attending clinics that had over 50 participants in HIV care in the programme, and if they were ART-naïve (by self-report) when enrolled into the programme. The participants in this cohort all began their treatment between 1^st ^February 2005 and 1^st ^June 2006, giving them an opportunity to have at least 10 months follow-up before the cohort was censored on the 31^st ^March 2007.

### Data sources and follow-up

The data were collected through routine health care services. Forms, completed at both scheduled and unscheduled visits, included information on CD4 count, haemoglobin, viral load, height, weight, and previous treatments. Participants with less than 8 g/mL of haemoglobin were classified as severely anaemic, and those with < 12 (<13 for men) g/mL as anaemic[11]. In this analysis, patients were followed up from the date of ART treatment initiation until the date of death, date of leaving the programme, lost to follow-up or 31^st ^March 2007, whichever came first. Deregistration forms provided information on participants leaving the programme alive and deaths whilst on treatment. Deaths were ascertained based on information from relatives or local hospital services, or by clinic staff attempting to contact patients who had not attended scheduled follow-up appointments. Records were checked to confirm that participants without a death or deregistration recorded were alive and attending the clinic as of 31^st ^March 2007. Reasons for leaving the programme, documented on the deregistration form, were relocated out of the area, transferred out of the programme or patient stopped treatment. Patients were defined as lost to follow-up if either the deregistration form documented lost to follow-up with no supplementary data for the reason or the most recent clinic visit occurred more than 6 months before 31^st ^March 2007.

### Statistical analysis

Data were analysed using STATA v. 10 (Stata Corporation, College Station, Texas, USA). CD4 count, viral load, haemoglobin levels and body mass index (BMI) at baseline were defined as the results closest to the date of ART start, restricted to the time interval 90 days before and 15 days after ART start, with viral load restricted to within 4 days after ART start, and if more than one measurement was available, the viral load before ART start was used.

The cohort was analysed using an intent-to-treat approach, assuming that once started on ART, patients remained on ART without interruption [7,12]. Mortality rates were compared using Poisson regression. Kaplan-Meier curves were used to summarise survival experience and Kaplan-Meier probabilities of survival at 3 and 9 months were also calculated for each variable.

Univariable analysis was carried out using the Cox proportional hazards model with shared frailty to calculate unadjusted hazard ratios for baseline variables. Shared frailty was used to take into account that individuals attending the same clinic may be more similar to each other with respect to outcomes and measures associated with outcomes than to those attending other clinics. Likelihood ratio tests were used to assess departures from linearity and for linear trend for ordinal variables. Proportional hazards were assessed by a lexis expansion at 3 months follow-up time and Schoenfeld residuals were also examined for departures from the proportional hazards assumption.

A multivariable model was constructed by retaining variables that were considered *a priori *confounders and those that were associated in univariable analysis (p < 0.1 based on likelihood ratio tests). BMI as a baseline risk factor had more than 20% missing data so a model using multiple imputation was examined, with 10 imputed datasets derived [13] and estimates combined on the basis of Rubin's Rules [14]. Baseline characteristics of patients who left the programme alive were compared with those who were still in follow-up at the censoring date for the cohort using the chi-square test or Wilcoxon rank sum test.

### Ethical considerations

This study was approved by the Research Ethics Committees of the University of KwaZulu-Natal, South Africa and the London School of Hygiene and Tropical Medicine, UK. Data were anonymised prior to analysis.

## Results

### Characteristics of study population

**Figure 1:**
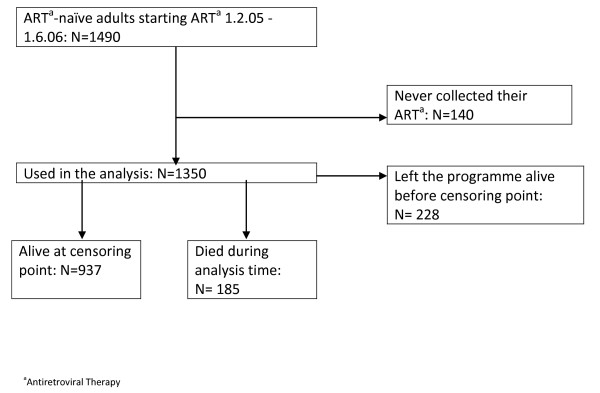
**Flow diagram describing antiretroviral therapy cohort inclusion and exclusion criteria and outcome at censoring**.

There were 1490 patients reported to be ART-naïve at the time of ART start between 1^st ^February 2005 and 1^st ^June 2006. Patients were excluded if they had never collected their medication (n = 140), hence there were 1350 included in the analysis (figure 1). These patients were from 27 clinics (16 in urban and 9 in peri-urban locations) and were situated in Gauteng (15 clinics), North West (6), Limpopo (2), Mpumalanga (2) and Free State (2) provinces.

The patients included in the analysis had a median age of 35.5 years (interquartile range [IQR] 30.2, 41.9) and 59.8% were female (table 1). At ART start the median CD4 count was 83 cells/μL, median haemoglobin level was 11.5 g/mL and the median viral load was 4.92 log_10 _copies/mL. The starting regimens included stavudine/lamivudine/efavirenz for 49.8%, stavudine/lamivudine/nevirapine for 41.6%, and zidovudine/lamivudine/efavirenz for 4.1%. The median BMI was 21.4 (kg/m^2^) and 8.7% were classified as severely anaemic (<8 g/mL) at baseline (table 1).

**Table 1:** Descriptive analysis of all antiretroviral therapy cohort participants (n = 1350)

Baseline characteristics	All patients (n = 1350)
Female	808 (59.8%)

Median age in years (IQR)	35.5 (30.2, 41.9)

CD4 count at baseline	n = 1257
Median number of cells per μL (IQR)	83 (27,147)
Count <50 cells per μL	473 (37.6%)

Viral load at baseline	n = 1187
Median log10 copies per mL (IQR)	4.92 (4.42, 5.38)
Count >5 log10 copies per mL	533 (44.9%)

Haemoglobin at baseline	n = 1206
Median g/mL (IQR)	11.5 (9.9, 13)
Count <8 g/mL (severely anaemic)	105 (8.7%)

Body mass index (BMI) at baseline	n = 854
Median kg/m^2 ^(IQR)	21.4 (18.5, 24.3)
Count <18.5 kg/m^2 ^(underweight)	207 (24.2%)

Previous tuberculosis diagnosis	314 (23.3%)

Current smoker	177 (13.1%)

Currently drinks alcohol	149 (11%)

Previous tuberculosis preventive therapy	30 (2.2%)

Treatment combination	
Stavudine/lamivudine/nevirapine	562 (41.6%)
Stavudine/lamivudine/efavirenz	672 (49.8%)
Zidovudine/lamivudine/efavirenz	55 (4.1%)
Other	61 (4.5%)

Footnote: IQR - Interquartile range;

### Outcomes and follow-up

The median follow-up time for the 1350 participants in this cohort was 13.4 months (IQR 10.1, 16.8) with 937 participants alive and in the programme at the censoring date of 31^st ^March 2007, and 185 deaths. A total of 228 participants were censored alive before 31^st ^March 2007; 148 (64.9%) patients were defined as lost to follow-up and 80 (35.1%) patients left the programme early (30 relocated out of the area, 26 stopped treatment and 24 transferred out of the programme). Those who were censored alive before 31^st ^March 2007 compared to those who were alive at the end of follow-up were more likely to be male (p = 0.01, chi-square test) and their median haemoglobin level was 11.4 g/mL compared to 11.8 g/mL (p = 0.03, Wilcoxon rank sum test); there was no evidence for differences between those who left the programme and those alive at the end of follow-up in terms of baseline CD4 count, BMI, and age at ART start (data not shown).

### Mortality rates

There were 185 deaths during follow-up giving an overall mortality rate of 13 per 100 person years (pyrs), with 94% alive at 3 months and 88% at 9 months. The mortality rate in the first 3 months was 24.5 per 100 pyrs, decreasing to 13 and 6 per 100 pyrs for 3 - 9 months and greater than 9 months following the start of ART, respectively (p < 0.0001). Seventy nine of the deaths (42.7%) occurred in the first 3 months.

### Risk factors for mortality

In univariable analysis using Cox regression with shared frailty, lower CD4 count, lower haemoglobin, low BMI and higher viral load, were all found to be associated with higher mortality (Table 2). The hazard ratios for participants with CD4 counts between 50 - 99 cells/μL, 100-200 cells/μL and >200 cells/μL compared to those with CD4 count<50 cells/μL were 0.37 [95% confidence interval (CI) (0.23, 0.58)], 0.28 [95% CI (0.18, 0.42) and 0.21 [95% CI (0.10, 0.45)] respectively. Proportional hazards were not satisfied for haemoglobin (p = 0.0002). There was no evidence of associations with hazard of mortality for ART regimen, previous tuberculosis diagnosis, gender, or age (Table 2).

**Table 2:** Unadjusted hazard ratios, 95% confidence intervals and survival probabilities for 3 and 9 months (n = 1350)

	Number of patients (Column %)	Unadjusted hazard ratio (95% CI)	Likelihood ratio test p-value	Kaplan-Meier probability of survival 3 months 9 months	
**Gender**			**p = 0.49**		
Male	542 (40.2%)	1		94%	86%
Female	808 (59.8%)	0.9 (0.67, 1.21)		94%	87%

**Age group (years)**			**p = 0.14**		
18 to 29	325 (24%)	1		95%	91%
30 to 34	314 (23.3%)	1.36 (86.7, 2.14)		96%	88%
35 to 39	288 (21.3%)	1.77 (1.14, 2.74)		92%	86%
40 to 49	307 (22.7%)	1.31 (0.83, 2.08)		94%	89%
50 to 70	116 (8.6%)	1.44 (0.79, 2.63)		94%	86%

**CD4 count (per μL) (n = 1257)**			**p < 0.0001**		
0 to 49	473 (37.6%)	1		87%	79%
50 to 99	241 (19.2%)	0.37 (0.23, 0.58)		97%	92%
100 to 200	414 (32.9%)	0.28 (0.18, 0.42)		98%	94%
> 200	129 (10.3%)	0.21 (0.10, 0.45)		97%	95%

**Viral load (log_10 _copies/mL) (n = 1187)**			**p < 0.0001**		
<4	134 (11.3%)	0.30 (0.15, 0.60)		98%	94%
4 to 5	520 (43.8%)	0.47 (0.34, 0.66)		97%	92%
>5	533 (44.9%)	1		90%	82%

Haemoglobin (g/mL) (n = 1206)*			**p < 0.0001**		
<8	105 (8.7%)	3.24 (2.12, 4.94)		79%	66%
8.1 - 9.9	207 (17.2%)	1.94 (1.32, 2.86)		87%	79%
10 - 11.9 (12.9 for men)	453 (37.6%)	1		96%	89%
>11.9 (>12.9 for men)	441 (36.5%)	0.38 (0.24, 0.63)		99%	96%

**Body mass index (kg/m^2^) (n = 854)**			**p < 0.0001**		
<18.5	207 (24.2%)	2.05 (1.41, 2.96)		88%	78%
18.5 to 25	471 (55.1%)	1		95%	88%
>25	176 (20.6%)	0.67 (0.38, 1.16)		99%	93%

**previous tuberculosis diagnosis**			**p = 0.12**		
Yes	314 (23.3%)	1.3 (0.94, 1.81)		94%	86%
No	1036 (76.7%)	1		94%	89%

**previous tuberculosis preventive therapy**					
Yes	30 (2.2%)	0.86 (0.31, 2.35)	**p = 0.77**	93%	93%
No	1320 (97.8%)	1		94%	88%

**Treatment combination**			**p = 0.41**		
Stavudine/lamivudine/nevirapine	562 (41.6%)	1		94%	89%
Stavudine/lamivudine/efavirenz	672 (49.8%)	0.91 (0.67, 1.24)		93%	88%
Zidovudine/lamivudine/efavirenz	55 (4.1%)	1.18 (0.57, 2.47)		98%	87%
Other	61 (4.5%)	0.52 (0.21, 1.29)		98%	91%

CI - Confidence Interval.

* Evidence against proportional hazards, p - value for interaction with follow-up time = 0.0002. Unadjusted hazard ratios (95% CI) in the first 3 months of ART for participants with haemoglobin <8, 8.1-9.9 and >11.9(f)/12.9 (m) g/mL were 3.24 (2.12, 4.94), 1.94 (1.32, 2.86) and 0.38 (0.24, 0.63) respectively comparing to 10-11.9 (f)/12.9 (m)g/mL, and for greater than 3 months of ART 2.02 (1.09, 3.74), 1.34 (0.77, 2.31), and 0.52 (0.31, 0.89), respectively.

**Figure 2:**
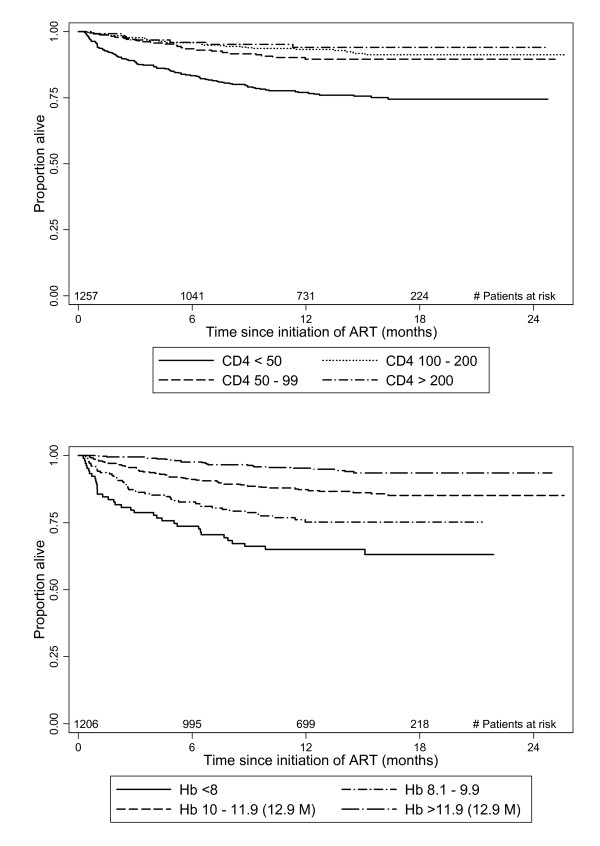
**Kaplan-Meier plots by CD4 count (μL) among 1257 adults starting antiretroviral therapy (top panel) and haemoglobin (g/mL) levels among 1206 adults starting antiretroviral therapy (bottom panel)**.

Kaplan-Meier curves show the survival experience for the whole cohort stratified by baseline CD4 count and baseline haemoglobin in Figure 2. These show that participants with CD4 count <50 cells/μL had a much poorer prognosis for survival than participants with CD4 count ≥ 50 cells/μL and that the survival experience for those in the highest three categories was very similar. There was much more of a graded effect for haemoglobin compared to CD4 count with a clear pattern of an increase in mortality with decreasing haemoglobin levels.

In multivariable analysis CD4 count and haemoglobin were independent risk factors for mortality (Table 3). After adjusting for age, gender, and haemoglobin, individuals with a CD4 count of <50 cells/μL were at much higher risk of mortality than those with ≥50 cells/μL, with an adjusted hazard ratio of 0.4 for CD4 count 50 - 99 cells/μL compared to those with <50 cells/μL. There was evidence that haemoglobin did not satisfy proportional hazards (p = 0.0003) with a stronger association of severe anaemia (<8 g/mL) on mortality in the first 3 months on ART (adjusted hazard ratio 4.99) compared with greater than 3 months on ART (adjusted hazard ratio 1.88; Table 3). Severe anaemia and low CD4 count identify different patients at higher risk of mortality: amongst patients with both CD4 count and haemoglobin, of those severely anaemic 28% (29/105) had CD4 count ≥100/μL whereas of those with haemoglobin >11.9 g/mL (>12.9 for men) one quarter (110/435) had CD4 count <50/μL. Viral load and BMI were not associated with mortality after controlling for haemoglobin and CD4 count.

**Table 3:** Adjusted hazard ratios for mortality from multivariate analysis, with 95% confidence intervals (n = 1195)

	Unadjusted HR^1 ^	aHR^2 ^(95% CI)	Likelihood Ratio test
			p-value
**Gender**			**0.41**
Male	1	1	
Female	0.89 (0.65, 1.23)	0.87 (0.63, 1.2)	

**Age group (years)**			**0.28**
18 to 29	1	1	
30 to 34	1.43 (0.88, 2.32)	1.37 (0.84, 2.22)	
35 to 39	1.85 (1.15, 2.97)	1.67 (1.03, 2.69)	
40 to 49	1.27 (0.78, 2.1)	1.25 (0.75, 2.07)	
50 to 70	1.44 (0.75, 2.74)	1.57 (0.82, 3.01)	

**CD4 count (/μL)**			**<0.0001**
0 to 49	1	1	
50 to 99	0.34 (0.21, 0.55)	0.40 (0.25, 0.64)	
100 to 200	0.29 (0.19, 0.45)	0.38 (0.25, 0.58)	
> 200	0.22 (0.1, 0.48)	0.34 (0.16, 0.74)	

**Haemoglobin (g/mL)**			**0.0003^3^**
*Time < 3 months*			
<8	5.35 (2.91, 9.84)	4.99 (2.69, 9.24)	
8.1 - 9.9	2.98 (1.67, 5.33)	3.05 (1.71, 5.48)	
10 - 11.9 (12.9 for men)	1	1	
>11.9 (>12.9 for men)	0.10 (0.23, 0.43)	0.12 (0.03, 0.52)	
*Time *≥ *3 months*			
<8	2.00 (1.10, 3.70)	1.88 (1.01, 3.49)	
8.1 - 9.9	1.27 (0.73, 2.20)	1.33 (0.77, 2.31)	
10 - 11.9 (12.9 for men)	1	1	
>11.9 (>12.9 for men)	0.50 (0.29, 0.86	0.59 (0.34, 1.02)	

^1 ^Unadjusted hazard ratio restricted to n = 1195 in model

^2^aHR (adjusted hazard ratio) adjusted for other variables in the table

^3 ^p-value for interaction with follow-up time.

There was less than 10% change in the adjusted hazard ratios for BMI from multiple imputation compared to the adjusted hazard ratios from the reduced multivariable model with no imputation, after controlling for haemoglobin and CD4 count. Evidence for BMI being associated with mortality was weak (p = 0.11) which is consistent with the conclusions from the multivariable analysis with no imputation.

## Discussion and Conclusions

This analysis, based on routinely collected data at clinics across South Africa, showed that there was high early mortality on ART with a large proportion (42.7%) of the deaths occurring in the first three months of treatment, consistent with other studies from sub-Saharan Africa [3]. This is most likely attributable to very advanced disease at treatment start as evidenced by low CD4 count at baseline, typical of clinics in sub-Saharan Africa [8,15-17]. High early on-ART mortality rates in resource-poor settings compared with industrialised countries are not completely explained by lower CD4 counts and more advanced clinical stage at baseline, but are probably compounded by co-morbidities, particularly tuberculosis, that are common in resource-poor settings[9]. Thus there is a need to identify individuals at high risk of death who may benefit from investigation for concurrent disease within a more intensive case management programme. This analysis indicates that low CD4 count and low haemoglobin are important independent prognostic factors for mortality in this setting and either or both could be used to identify those at highest risk of death.

At three months after ART start, 13% of those with a baseline CD4 count below 50 had died compared with 3% of those with CD4 count of 50 and above, highlighting the strong association between baseline CD4 count and early mortality, consistent with other studies [3,5,7,12,18,19]. Our results are also consistent with other studies showing that anaemia predicts mortality among individuals on ART in Africa and developed countries [12,18,20-22] although evidence is limited and thus this analysis, from a large cohort receiving care at multiple clinics, adds valuable information to support this. Anaemia in these settings is associated with conditions such as tuberculosis [Hanifa Y, unpublished data, personal communication] which may not be clinically apparent, but could potentially be treated; as well as other co-morbidities such as malnutrition, gastrointestinal Kaposi's sarcoma etc [19]. Thus those individuals starting ART with a low haemoglobin in these settings are in particular need of investigation for, and if necessary treatment of, tuberculosis. More generally haemoglobin levels could be used by clinics to identify more promptly participants that are at very high risk of mortality in ART programmes when they start treatment and be very useful in settings where CD4 counts are less widely available. There could then be a programme of intensified case management and monitoring for these individuals at high risk. This could also involve more investigation for co-morbidities especially tuberculosis but should not delay the start of ART as there is a very high mortality rate for those waiting to start ART [5].

One in six participants in this cohort left the programme during follow-up, and for the majority of these there was none or only limited information on reasons for their departure, similar to other ART programmes in Africa [12,15,23,24]. Although these individuals were similar to those alive at the end of follow-up in age, gender, CD4 count, viral load and BMI, the loss to follow-up may also lead to an under-estimate of mortality rates for this cohort as an unknown number of the 11.0% participants lost to follow-up may have died soon after leaving the programme. Studies in similar settings have shown that mortality is often underestimated when individuals are lost to follow-up and not actively traced [9,25-27].

A strength of the data presented in this analysis is that individuals identified in this cohort had definitely started ART and collected their medication. This helps to reduce the bias that may occur if individuals who never started ART were contributing person-years to the study.

There were missing data for BMI (36.7%) but it was evaluated in the univariable and multivariable analyses and conclusions from the multivariable model were supported by multiple imputation methods. There was also no information on the cause of death for this cohort which would have been useful, as what had caused the early deaths and information regarding the deaths of those with very low haemoglobin would help to inform an intensified case management system. However there is increasing evidence from other studies about the importance of tuberculosis as an early cause of death [28,29]

The results from this analysis give evidence that CD4 count and haemoglobin predict early mortality for individuals on ART in sub-Saharan Africa. Individuals starting ART with either low CD4 count or low haemoglobin should be targeted for intensified case management, including investigation for and treatment of coexistent infections, particularly tuberculosis in settings of high tuberculosis prevalence. In clinics without access to CD4 counts, haemoglobin may be useful to identify those at highest risk.

## Competing interests

The authors declare that they have no competing interests.

## Authors' contributions

ECR, SC, ADG, KF designed the study; SC & LP collected the data; ECR, KF carried out data analysis and interpretation; ADG, SC and GJC carried out data interpretation; ECR, SC, ADG, KF drafted the manuscript; GJC & LP revised the manuscript. All authors have read and approved the final version of the manuscript.

## Pre-publication history

The pre-publication history for this paper can be accessed here:

http://www.biomedcentral.com/1471-2458/10/433/prepub
